# Exploring the impact and influence of melanin on frequency-domain near-infrared spectroscopy measurements

**DOI:** 10.1117/1.JBO.29.S3.S33310

**Published:** 2024-09-25

**Authors:** Shidhartho Roy, Jingyi Wu, Jiaming Cao, Joel Disu, Sharadhi Bharadwaj, Elizabeth Meinert-Spyker, Pulkit Grover, Jana M. Kainerstorfer, Sossena Wood

**Affiliations:** a Carnegie Mellon University, Department of Biomedical Engineering, Pittsburgh, Pennsylvania, United States; b Carnegie Mellon University, Department of Electrical and Computer Engineering, Pittsburgh, Pennsylvania, United States; c Carnegie Mellon University, Neuroscience Institute, Pittsburgh, Pennsylvania, United States

**Keywords:** near-infrared spectroscopy, melanin index, signal-to-noise ratio, oxygen saturation, optical properties

## Abstract

**Significance:**

Near-infrared spectroscopy (NIRS) is a non-invasive optical method that measures changes in hemoglobin concentration and oxygenation. The measured light intensity is susceptible to reduced signal quality due to the presence of melanin.

**Aim:**

We quantify the influence of melanin concentration on NIRS measurements taken with a frequency-domain near-infrared spectroscopy system using 690 and 830 nm.

**Approach:**

Using a forehead NIRS probe, we measured 35 healthy participants and investigated the correlation between melanin concentration indices, which were determined using a colorimeter, and several key metrics from the NIRS signal. These metrics include signal-to-noise ratio (SNR), two measurements of oxygen saturation (arterial oxygen saturation, ${\mathrm{SpO}}_{2}$, and tissue oxygen saturation, ${\mathrm{StO}}_{2}$), and optical properties represented by the absorption coefficient ($\mu_{a}$) and the reduced scattering coefficient ($\mu_{s}^{\prime }$).

**Results:**

We found a significant negative correlation between the melanin index and the SNR estimated in oxy-hemoglobin signals ($r_{s}=-0.489$, p=0.006) and ${\mathrm{SpO}}_{2}$ levels ($r_{s}=-0.413$, p=0.023). However, no significant changes were observed in the optical properties and ${\mathrm{StO}}_{2}$ ($r_{s}=-0.146$, p=0.44).

**Conclusions:**

We found that estimated SNR and ${\mathrm{SpO}}_{2}$ values show a significant decline and dependence on the melanin index, whereas ${\mathrm{StO}}_{2}$ and optical properties do not show any correlation with the melanin index.

## 1 Introduction

Near-infrared spectroscopy (NIRS) is an optical method that uses light–tissue interaction to measure optical properties and characteristics. It is sensitive to changes brought on by variations in hemoglobin concentration and oxygenation, the latter referring to the level of oxygen in the blood. NIRS operates on the principle of changes in light intensity due to the absorption of hemoglobin and scattering in the imaged tissue. This sensitivity to light intensity is accomplished using at least two or more wavelengths, primarily within an optical window of 650 to 900 nm,^1^^,^^2^ where absorption spectra of oxyhemoglobin, deoxyhemoglobin, water, and melanin are present. The promise of portability and appropriateness for continuous bedside monitoring has propelled NIRS to its growing popularity in various domains, ranging from clinical studies in adult populations,^3^^,^^4^ multi-modal recording,^5^^,^^6^ cognitive research,^7^^,^^8^ and pediatric populations^9^^,^^10^ to exercise monitoring.^11^^,^^12^ Although NIRS and other diffuse optical methods are well-established imaging techniques,^13^^,^^14^ their application tends to produce reliable and consistent results for group-level analysis but may vary for single subject-level analysis due to inherent variability. This variability may be partly attributed to differences in melanin, a primary determinant of skin color and the product of melanocytes in the skin’s basal layer.^15^^–^^17^

There is a significant concern regarding the effect of melanin on NIRS measurements and participant variability across skin tones in the existing literature and regulatory affairs as melanin has the potential to reduce light intensity,^18^ light sensitivity, and signal-to-noise ratio (SNR).^19^^,^^20^ Recently, the Food and Drug Administration (FDA) has addressed concerns in its virtual public meetings of the Center for Devices and Radiological Health about several studies reporting that pulse oximeters, which have a similar principle but different function from NIRS, may provide less accurate readings for individuals with darker skin pigmentation. Although early research suggested that the effect of melanin on NIRS measurements was not significant,^21^^,^^22^ emerging evidence reveals that melanin has a more severe impact on the measurement of scattering and absorption,^23^ the reflected NIRS signal,^24^ SNR,^25^ and measurements of oxygenation.^26^^,^^27^ Bozkurt et al.^28^ showed that as the melanin concentration of a participant does not change during measurement, it did not greatly impact the hemoglobin concentration changes. Yet, they reported that increased melanin concentration could necessitate higher light intensity for achieving an acceptable SNR in participants with darker skin.^28^ In the NIR range, obtaining higher light intensity is challenging due to the significant overlap of absorption spectra, cross-talk, and variations in melanin levels among participants. To address this challenge, Lloyd et al.^29^ demonstrated the need to increase source power levels when studying individuals with darker skin due to stronger light attenuation, highlighting the role that melanin concentration plays in influencing NIRS signal quality.^27^^,^^30^ To support this finding, Althobaiti et al.^25^ found a decrease in SNR for different wavelengths ranging from 450 to 1050 nm. Their Monte Carlo simulation modeled differences in skin layers, explicitly accounting for blood content, water content, and melanin concentration in the epidermis. The simulation provided insights into the sensitivities of both the epidermis and dermis layers, offering insight into light–tissue interactions in the context of varying melanin. Althobaiti et al. demonstrated the need to isolate the contribution of oxyhemoglobin, deoxyhemoglobin, and melanin to NIRS data. Regrettably, many NIRS studies did not report ethnicity, race, or classifications of melanin concentration within their participant groups, leaving an essential factor and an explanation of reduced light intensity and sensitivity unaccounted for and reported during data collection.^31^^–^^33^

To address this gap, some researchers have categorized participant melanin concentration in NIRS studies by self-reported skin-type scales, race, ethnicity, or direct melanin measurements using colorimeters to highlight the importance of diversifying datasets and addressing the biases of earlier literature.^34^ Self-reported skin-type scales, such as the Fitzpatrick scale, can be inaccurate.^35^^,^^36^ Categorizations of race and ethnicity challenge the reliability of conclusions due to the limitation of the variability within racial categories and the geographical participant pools. Colorimeters have helped improve measurements of melanin accuracy but do not address all limitations. For example, Dubois et al.^37^ revealed the complexities of correlating melanin levels to NIRS measurements, pointing out the influence of other pigment phenotypic features such as hair color, texture, and curl pattern. Dubois et al. particularly underscored the need to enhance inclusive participant recruitment practices to reflect and address racial biases in research studies more accurately and to fairly encompass a wider melanin index range beyond small sample sizes.

The signal quality can also be manifested in multiple metrics, such as tissue oxygenation (${\mathrm{StO}}_{2}$). For ${\mathrm{StO}}_{2}$, multiple studies have focused on race for cerebral oximetry.^27^ Couch et al.^34^ showed that there is a negative correlation between muscle ${\mathrm{StO}}_{2}$ measurements with melanin index reported from three different devices. Similarly, Afshari et al.^26^ reported a negative correlation of ${\mathrm{StO}}_{2}$ values as melanin concentrations increase in epidermis-simulating phantom with a cerebrovascular module. Sun et al.^27^ reported lower ${\mathrm{StO}}_{2}$ values among African Americans than Caucasians. To date, most studies reporting on the effects of melanin impact on arterial oxygenation, ${\mathrm{SpO}}_{2}$, have come from pulse oximetry, which operates on the same principles as NIRS but typically is measured as a transmitted signal through the finger and is influenced by the presence of melanin.^38^

A consensus among various studies indicates that the device measurement bias escalates with lower arterial oxygen saturation values, a phenomenon largely attributed to the underrepresentation of certain patient groups during the calibration process of these devices.^15^^,^^17^^,^^39^^,^^40^ Patients with darker melanin were reported to have a three times higher (statistically significant) risk of occult hypoxemia.^41^^,^^42^ In response to these findings, there have been several propositions for policy^43^ and technological changes^44^ aimed at mitigating racial bias in pulse oximetry. Because of the complexities of this limitation in NIRS and reporting melanin concentration, a more thorough investigation is required to determine the quantitative effect of melanin on NIRS measurements. In this study, we address three research questions that address the limitation in NIRS and reporting melanin concentration:

1. How do the inclusion of a diverse participant pool and quantification of melanin impact the accuracy and generalizability of findings in NIRS-based metrics compared with current NIRS practices?
2. Is there an impact of melanin concentration on commonly used NIRS measurement metrics that differ when applying the modified Beer–Lambert law to obtain relative changes in absorption as opposed to more complex analyses of FD-NIRS?
3. Do the fundamental principles of FD-NIRS devices help provide information regarding any observed discrepancies in the NIRS signal due to melanin?

## 2 Material and Methods

### 2.1 Participant Details

**Fig. 1:**
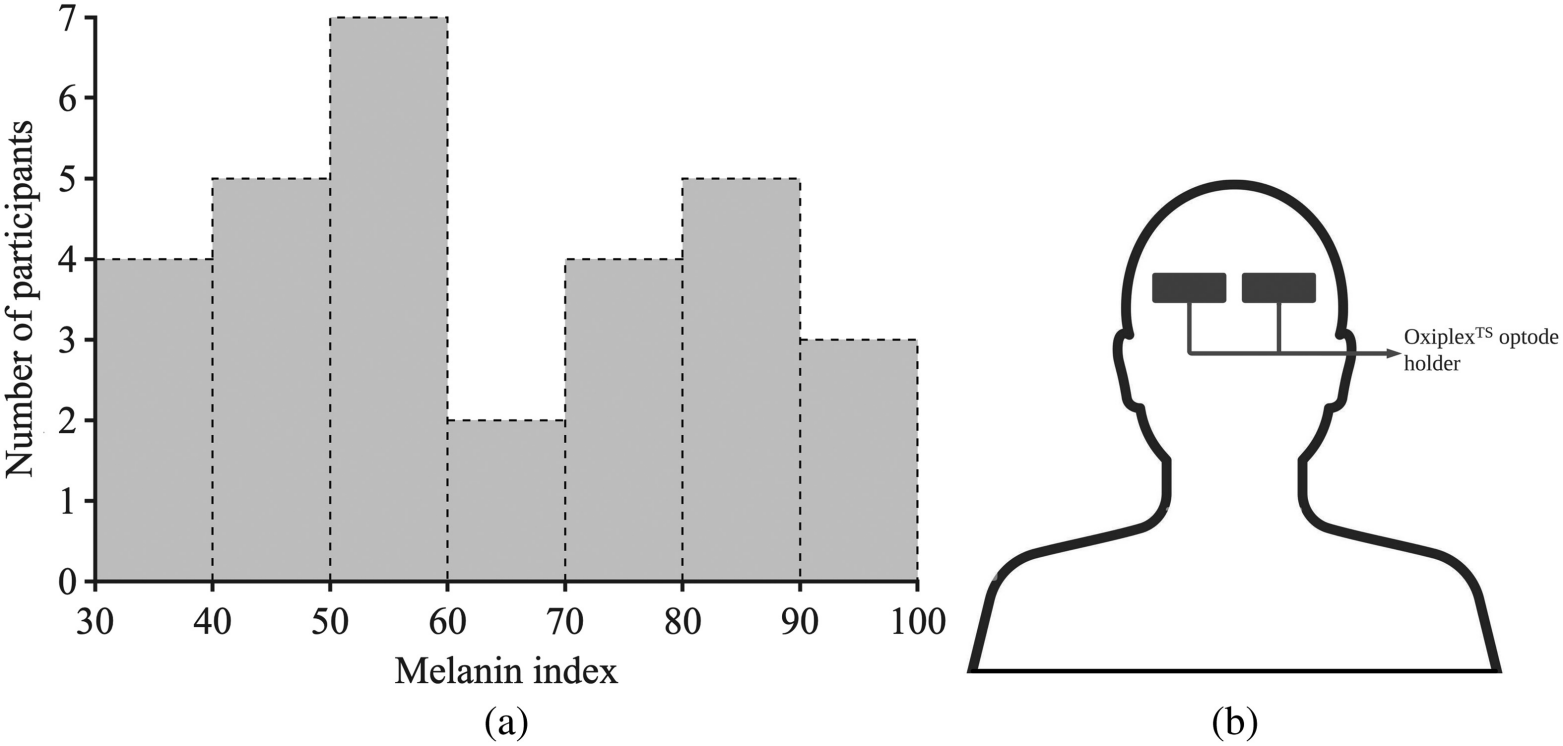
Participant distribution according to the melanin level is shown in panel (a). The melanin index is measured with DSM−III. Panel (b) shows the optode placement in the forehead.

A total of 35 healthy adult participants without pre-existing medical conditions were recruited. Participants ranged from 18 to 30 years old with varying levels of melanin [Fig. 1(a)]. All participants were enrolled in the study in accordance with the Institutional Review Board guidelines. Both verbal and written informed consent was obtained from each participant. Data from five participants were excluded due to motion artifacts or technical issues during the data collection.

### 2.2 Instrumentation

We used an FD-NIRS system (ISS Oxiplex™, Champaign, Illinois, United States) to acquire amplitude and phase of intensity modulated light at four source-detector distances. A version of the ISS Oxiplex™ device has recently received FDA approval for clinical use, marking the first approval for any device in this category. This instrument operates at a modulation frequency of 110 MHz and utilizes two wavelengths: 690 and 830 nm. Data were sampled at a frequency of 50 Hz. Optical probes were placed on the forehead, as illustrated in Fig. 1(b), and source-detector distances were 20, 25, 30, and 35 mm. To quantify the melanin content of each participant, we measured the melanin index using a DSM-III colorimeter (Cortex Technology ApS, Aalborg, Denmark), a non-invasive device that measures melanin via reflective spectroscopy^45^ location as the optical probe. It is important to note that our DSM-III colorimeter cannot directly measure the individual typology angle (ITA) in coherence with the FDA’s recent suggestion for an objective measurement of skin pigmentation.^46^ Nevertheless, we found ITA and melanin index to be interchangeable (as seen in Fig. S2 in the Supplementary Material).

The experimental setup began with each participant comfortably seated in a controlled environment with minimum ambient light. The built-in automatic gain optimization function was used to optimize the detector gain. Resting-state data were collected for 3 min, from which a continuous 60-s segment of resting-state data was chosen for each participant with minimal heart rate variability. The 60-s segment of resting-state selection was based on a visual inspection of the time traces, with the goal of identifying a time segment demonstrating minimal motion artifact presence for the specified duration. Any sudden and large shifts in baseline measurements of relative and absolute measurements of oxyhemoglobin and deoxyhemoglobin were deemed indicative of motion presence. Notably, no motion artifact correction algorithm was employed in this process.

### 2.3 SNR Estimation Analysis

To quantify the impact of melanin concentration on commonly used NIRS measurement metrics, we report four metrics—SNR estimation, arterial and tissue oxygen saturation values, and optical properties. We used the modified Beer–Lambert law (mBLL) to calculate changes in oxyhemoglobin and deoxyhemoglobin concentrations.^47^ The differential pathlength factor ratio has been calculated based on the work of Ref. 48 (the details are available in Fig. S1 in the Supplementary Material). The changes in concentrations of oxygenated and deoxygenated hemoglobin (Δ[HbO] and Δ[Hb], respectively) can be expressed in micromolar (μM) units and are calculated using the following expression:

$$\Delta \mu_{a}^{\lambda }=-\frac{1}{r.\mathrm{DPF}}\cdot \ln(\frac{I^{\lambda }}{I_{0}^{\lambda }}).$$ (1)

The extinction coefficient for calculating Δ[HbO] and Δ[Hb] quantifies the degree to which a substance absorbs light at a specific wavelength.^49^ We used the frequency domain multiple-distance (FDMD) method to convert the amplitude and phase of the measured intensities as a function of distance into absolute measures of hemoglobin concentration and oxygenation using the following equation:^50^

$$\mu_{a}=\frac{\omega }{2\nu }(\frac{S_{\varphi }}{S_{\mathrm{AC}}}-\frac{S_{\mathrm{AC}}}{S_{\varphi }}),\;\mu_{s}^{\prime }=\frac{S_{\mathrm{AC}}^{2}-S_{\varphi }^{2}}{3\mu_{a}}-\mu_{a}.$$ (2)

The SNR estimation analysis was primarily driven by the first harmonic of the cardiac waveform captured, which serves as an essential indicator of optimal optical contact. We chose the first harmonic of the cardiac waveform because it is most prominent and visible in all participants. This focus played a crucial role in guaranteeing the reliability and accuracy of the acquired data. Established SNR estimation methods^51^^–^^53^ have used cardiac pulsation to quantify SNR because the presence of cardiac pulsation is very robust in NIRS signals. Similarly, in our study, we defined the signal as the fundamental cardiac pulsation (Fig. S3 in the Supplementary Material). To accommodate heart rate variability, a power spectrogram was plotted utilizing a moving window of 20 s, with a 95% overlap. The maximum time-frequency point was then derived using the “tfridge” function of Matlab^54^ with a penalty of 0.005. The noise was defined as mean spectral power within 11 to 19 Hz to exclude harmonics and aliasing effects because there is an absence of physiological data and the potential for aliasing noise from electrical devices. Given these definitions of signal and noise, the SNR was estimated using the following equation:

$$\mathrm{SNR}=10\times \log(\frac{\text{Spectral power of the signal at cardiac pulse}}{\text{Spectral power of the noise}}).$$ (3)

This SNR estimation method provides a realistic assessment of signal strength versus background noise, assuming that the melanin’s effect is frequency-independent.

### 2.4 Assessment of Oxygen Saturation

We evaluated ${\mathrm{StO}}_{2}$ and ${\mathrm{SpO}}_{2}$ as a function of melanin index, where ${\mathrm{StO}}_{2}$, ${\mathrm{StO}}_{2}=[\mathrm{HbO}]/[\mathrm{HbT}]$. The arterial oxygen saturation, ${\mathrm{SpO}}_{2}$, was calculated using the following equation with respect to the heart rate:

$${\mathrm{SpO}}_{2}=\frac{\Delta [\mathrm{HbO}{]}_{\mathrm{HR}}}{\Delta [\mathrm{Hb}{]}_{\mathrm{HR}}+\Delta [\mathrm{HbO}{]}_{\mathrm{HR}}}.$$ (4)

Here, $\Delta [\mathrm{HbO}{]}_{\mathrm{HR}}$ and $\Delta [\mathrm{Hb}{]}_{\mathrm{HR}}$ denote the relative changes in oscillations of oxygenated and deoxygenated hemoglobin at the heart rate, respectively. To enhance the robustness against noise interference inherent in peak-finding algorithms, ${\mathrm{SpO}}_{2}$ was also estimated by their corresponding spectral powers at the heart rate, ascertained from the spectrogram Fig. S3(a) (for $\Delta [\mathrm{HbO}{]}_{\mathrm{HR}}$) and S3(b) (for $\Delta [\mathrm{Hb}{]}_{\mathrm{HR}}$) in the Supplementary Material. We determined the mean and standard deviation from a 60-s window of resting-state data. The spectrogram window spanned 20 s, with a 95% overlap.

Another equivalent method to calculate ${\mathrm{SpO}}_{2}$ applies the calibration curve (detailed in the Supplementary Material) from the ratio (R) as commonly done in pulse oximetry. With this expression, we can isolate the contribution of two wavelengths in ${\mathrm{SpO}}_{2}$ and become clearer to determine the relationship between R and ${\mathrm{SpO}}_{2}$. R is calculated from AC (pulsating component of the FD-NIRS intensity data) and DC (non-pulsating component of the FD-NIRS data). As our study does not include arterial blood gas measurements, all participants are assumed to maintain ${\mathrm{SpO}}_{2}$ levels close to 100%.

### 2.5 Statistical Analysis

We adopted a two-phased approach using Spearman’s rank correlation coefficient and linear regression model to analyze all investigated metrics. In the first phase, we calculated Spearman’s rank correlation coefficient, $r_{s}$, and the corresponding p-value of each metric where a p-value less than 0.05 is statistically significant. This non-parametric measure of rank correlation was explicitly chosen due to its ability to assess both linear and monotonic relationships, offering greater flexibility over the Pearson correlation coefficient.

The second phase of our analysis involved fitting a linear model to the measured dataset. The outcomes of this linear fit, namely, the coefficient of determination ($R^{2}$) and the slope of the fit, provide insights into the relationship between the metric under consideration and the melanin index. The $R^{2}$ value quantifies the proportion of variance in the dependent variable that can be predicted from the independent variable, offering a measure of explanatory power for the model. Conversely, the slope conveys the direction and rate of change in the metric with respect to changes in the melanin index. Together, these statistical methods yield a comprehensive understanding of the influence of the melanin index on the investigated metrics for the collected sample size. All analyses were performed with MATLAB (version R2022b, MathWorks, Natick, Massachusetts, United States).

## 3 Result

We found that self-reported race can fall within multiple melanin index bins using the colorimeter, and melanin indices vary across different races, as shown in Fig. S4 in the Supplementary Material. Our findings show that when the participants are evenly split by the melanin index, the SNR estimation decreases (slope=−0.445) and becomes more pronounced ($r_{s}=-0.464$, p=0.083) when the melanin index goes beyond 56 (Fig. S5 in the Supplementary Material). We further observed that the Fitzpatrick scale can be misleading in our participant group as the Fitzpatrick scale can have multiple melanin indices illustrated in Fig. S6 in the Supplementary Material.

**Fig. 2:**
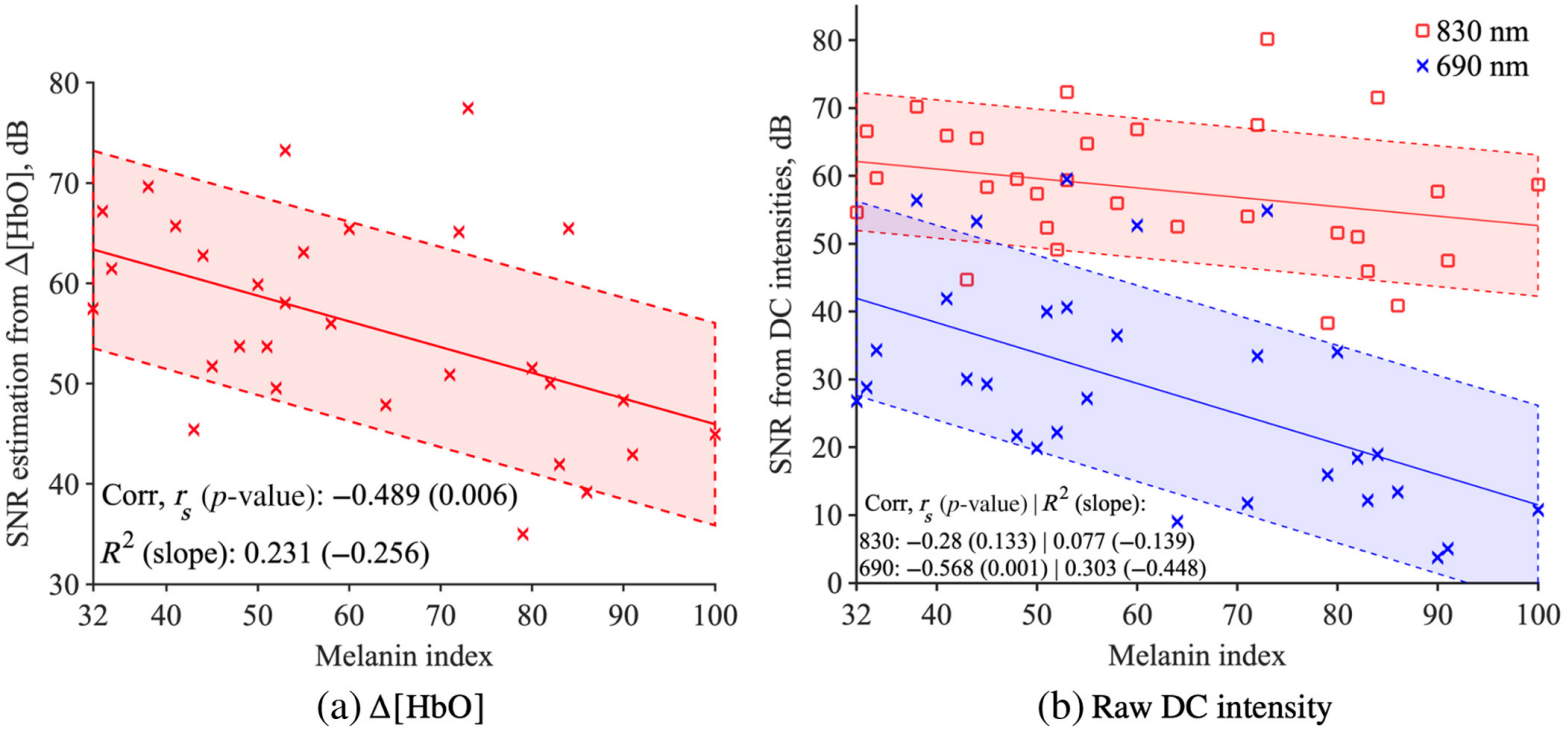
In both cases, SNR estimation shows a negative correlation, where panel (a) shows the correlation from Δ[HbO] signal is significant ($r_{s}=-0.489$, p=0.006) and panel (b) shows that for the 690 nm DC intensity, the correlation is higher, and for 830 nm (830 nm: $r_{s}=-0.28$, p=0.133), DC intensity the correlation is not significant (690 nm: $r_{s}=-0.568$, p=0.001).

A statistically significant negative correlation between SNR estimation based on the Δ[HbO] signal and melanin index was found ($r_{s}=-0.489$, p=0.006), as seen in Fig. 2(a), indicating that higher melanin indices are associated with lower SNR values. In the figures, the solid lines represent the data points’ linear fitting, and the shaded region enclosed by the dotted lines indicates the 95% confidence interval. The increase in noise mainly causes this decline in SNR estimation, Fig. S7 in the Supplementary Material ($r_{s}=0.380$, p=0.038), not by signal Fig. S8 in the Supplementary Material ($r_{s}=0.057$, p=0.765). This increasing noise does not significantly affect the ability to detect fundamental cardiac pulses in the Δ[HbO] signal at varying levels of the melanin index.

**Fig. 3:**
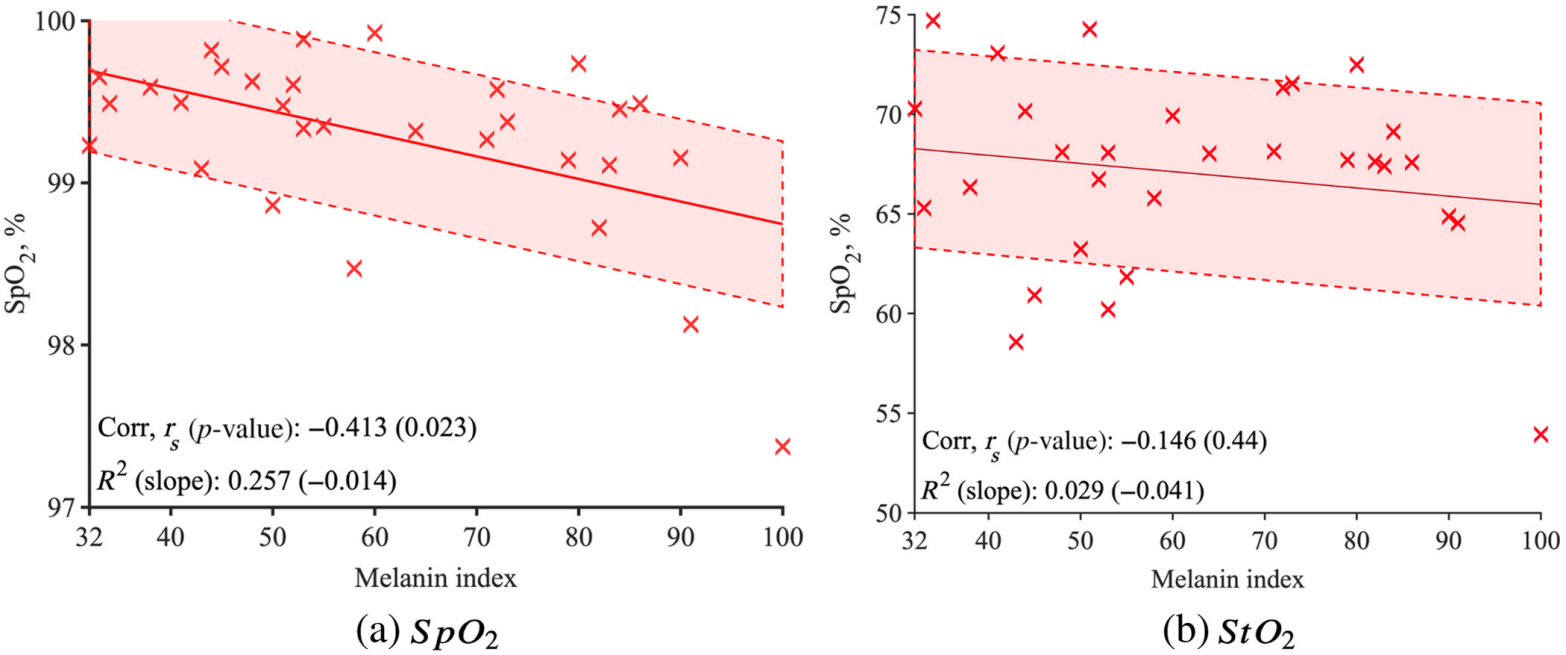
(a) ${\mathrm{SpO}}_{2}$ shows a significant negative correlation with the melanin index ($r_{s}=-0.146$, p=0.44), but (b) ${\mathrm{StO}}_{2}$ shows no statistically significant correlation ($r_{s}=-0.413$, p=0.023).

**Fig. 4:**
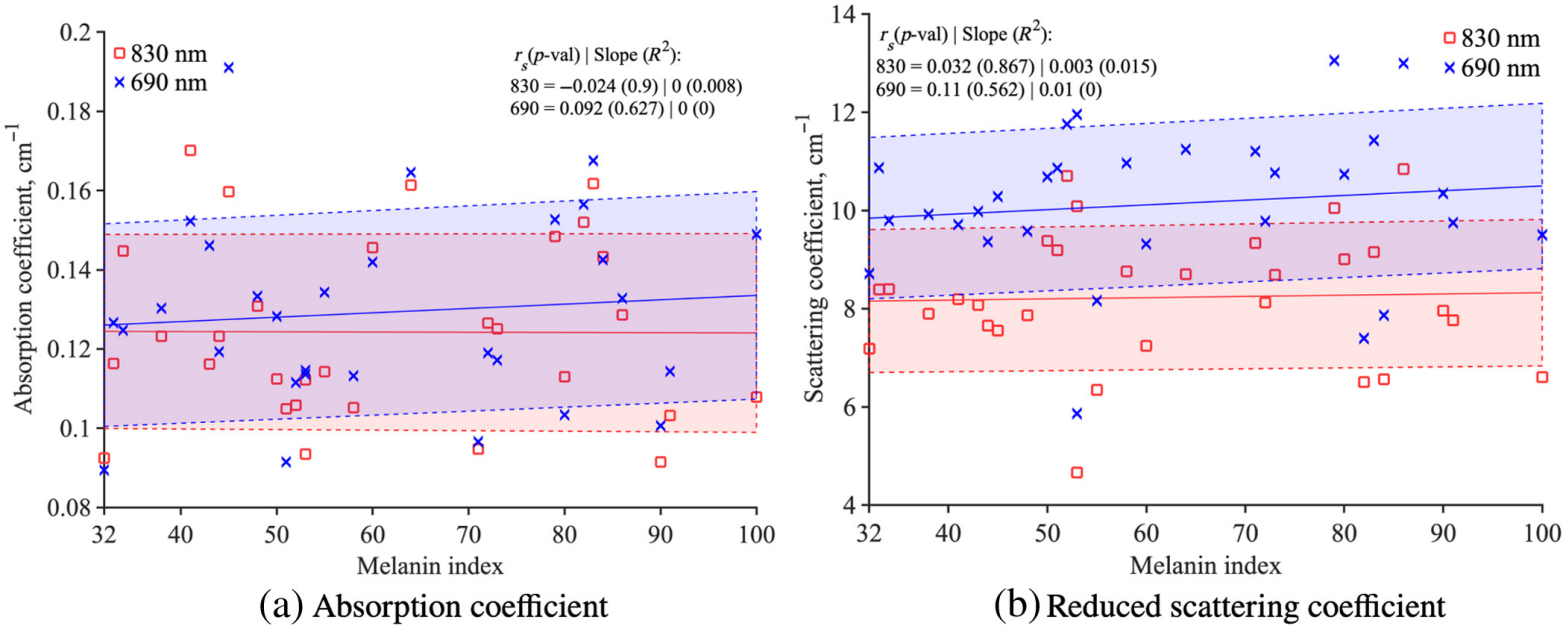
Both (a) $\mu_{a}$ (830: $r_{s}=-0.024$, p=0.9, 690: $r_{s}=0.092$, p=0.627) and (b) $\mu_{s}^{\prime }$ (830: $r_{s}=-0.032$, p=0.867, 690: $r_{s}=0.11$, p=0.562) do not show any significant correlation with the melanin index.

To understand further why the SNR estimation value decreases in Fig. 2(a), Fig. 2(b) shows SNR estimation for each wavelength. The SNR estimation was calculated using the same method as the SNR estimation from Δ[HbO] (described in Sec. 2.3). As the cardiac pulsation is more pronounced at 830 nm, the SNR estimation is higher for 830 nm than 690 mm. Both wavelength’s SNR estimations are decreasing with the melanin index, yet the 690 nm DC intensity shows that its SNR estimation decrease is statistically significant (830 nm: $r_{s}=-0.28$, p=0.133; 690 nm: $r_{s}=-0.568$, p=0.001). A statistically significant negative correlation was observed between ${\mathrm{SpO}}_{2}$ levels and melanin index ($r_{s}=-0.413$, p=0.023) and highlighted in Fig. 3(a). There is a low negative correlation between the ${\mathrm{StO}}_{2}$ value and the melanin index, but it is not significant [$r_{s}=-0.146$, p=0.44, Fig. 3(b)]. Absorption coefficient, $\mu_{a}$, [Fig. 4(a)] and reduced scattering coefficient, $\mu_{s}^{\prime }$ , [Fig. 4(b)] do not show a significant correlation with the melanin index.

The R value, according to Eq. (S1) in the Supplementary Material, against the melanin index is plotted in Fig. S9 in the Supplementary Material and shows a significant increase with the melanin index ($r_{s}=0.488$, p=0.006). To understand the components of R [Eq. (S2) in the Supplementary Material], both DC and AC components were analyzed at wavelengths of 830 and 690 nm. Both DC 830 and 690 nm significantly correlate with the melanin index. DC 830 nm has a positive correlation ($r_{s}=0.506$, p=0.004), and 690 nm has a negative correlation ($r_{s}=-0.491$, p=0.006), as seen in Fig. S10 in the Supplementary Material. On the other hand, the AC 690 has a significant negative correlation ($r_{s}=-0.374$, p=0.041) but not AC 830 nm ($r_{s}=0.073$, p=0.702; Fig. S11 in the Supplementary Material).

**Fig. 5:**
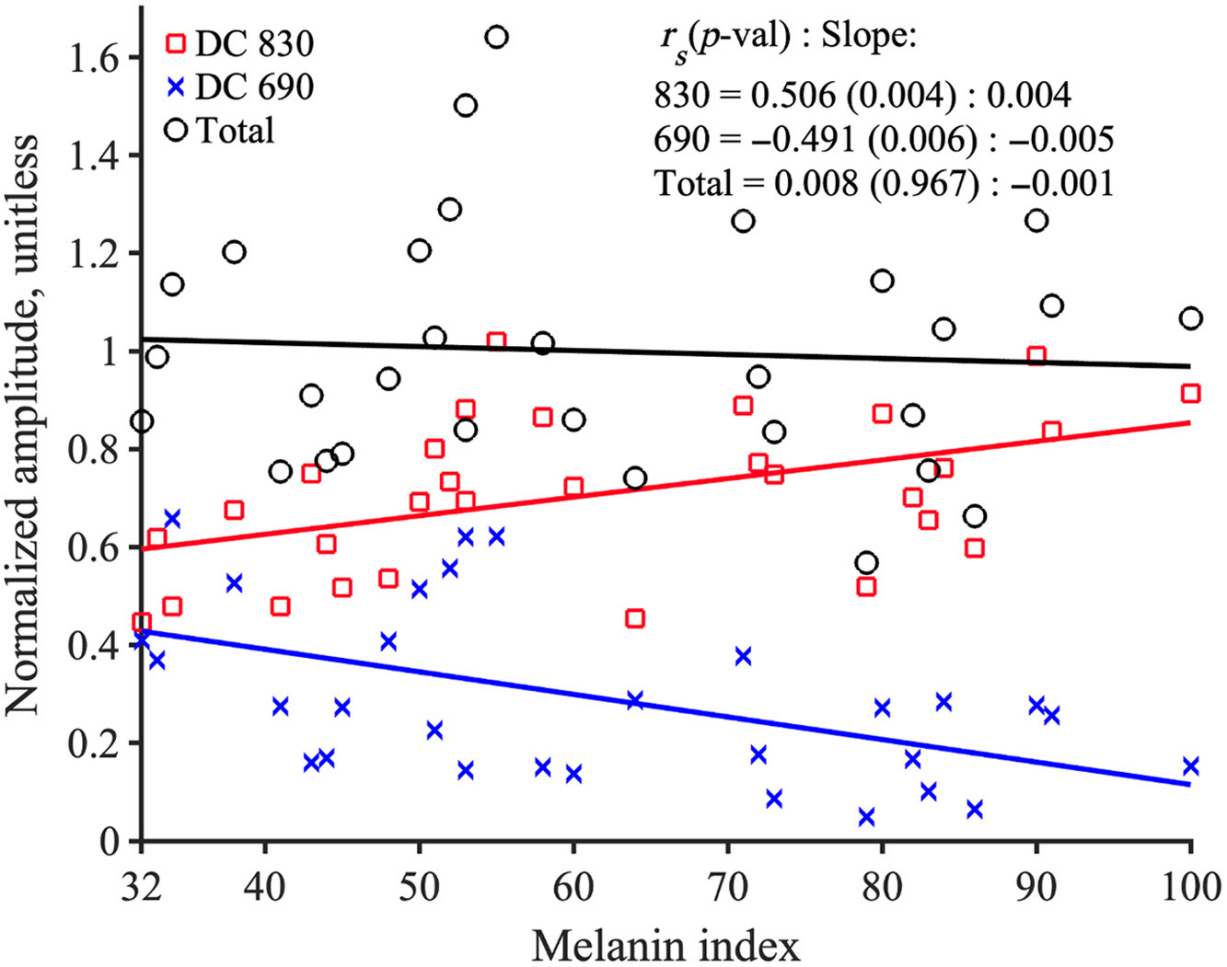
Total photon count number does not change ($r_{s}=-0.008$, p=0.967), but the 830 nm photon count increases ($r_{s}=0.506$, p=0.004), and the 690 nm photon count decreases significantly ($r_{s}=-0.491$, p=0.006).

In addition, it is important to note that the commercial FD-NIRS system uses gain settings to optimize the intensity for both wavelengths. The total photon count, as seen in Fig. 5, does not change with the melanin index ($r_{s}=0.008$, p=0.967). However, we found that the photon count is wavelength dependent, with 830 nm intensity increasing and the 690 nm intensity decreasing significantly.

## 4 Discussion

### 4.1 Research Question 1

In this study, we employed a colorimeter to quantify melanin by reporting a melanin index that addresses the limitations of prior research on melanin reporting. These studies predominantly focused on quantifying the effects of melanin on NIRS and other optical technologies by categorizing participants according to the Fitzpatrick scale, race, and ethnicity.^17^^,^^27^^,^^55^ However, these studies had challenges in reporting the wide variation in melanin that the Fitzpatrick scale, race, and ethnicity cannot properly report. We observed that the Fitzpatrick scale does not accurately correspond to consistent melanin indices in the races and ethnicities of our sampled population as there is a wide range of melanin indices, Fig. S6 in the Supplementary Material). This wide range of skin tones has been described as a limitation of the Fitzpatrick scale in previous studies^34^^,^^56^ and is further supported by our findings, as illustrated in Figs. S4 and S6 in the Supplementary Material). Our findings suggest that utilizing a colorimeter may help future NIRS studies address the variability of NIRS measurements in participants, especially as melanin increases. Although the Monk Skin Tone (MST) scale^57^ or the Fitzpatrick scale^58^ may be useful for diversifying recruitment of participants in research studies, these scales are not useful for the accurate quantitative performance of NIRS and pulse oximeters.

Furthermore, our findings highlight that the inclusion of colorimeter-derived melanin indices in these studies provides a critical method to improve the understanding of data diversity between Fitzpatrick scale categorization with a wide variety of melanin such as Fitzpatrick skin types IV to VI. Most studies have limitations because they did not recruit or report any or enough participants below a melanin index of 60 or Fitzpatrick skin type IV.^37^ In contrast, our study reveals that biases become more pronounced with melanin index values exceeding 56 [before melanin index 56 (slope: −0.170, p=0.704) and after 56 (slope value is −0.445, p=0.083), Fig. S5 in the Supplementary Material]. Therefore, more inclusive data collection strategies are imperative to comprehensively understand research analysis in various demographics. By incorporating a colorimeter in the research protocol, we can improve current NIRS practices and interpretation of the generalizability of findings in NIRS-based metrics in participants with darker melanin concentrations.

### 4.2 Research Question 2

In this study, we found that the SNR and ${\mathrm{SpO}}_{2}$ are significantly impacted by melanin, which is derived from the mBLL. We found that Δ[HbO] and Δ[Hb]. Δ[HbO] [Fig. 2(a)] and derived from the mBLL are significantly impacted by melanin. Our ${\mathrm{SpO}}_{2}$ analysis in Fig. 3(a) demonstrates further evidence that the wavelength dependency of melanin absorption has significantly impacted the SNR estimation and ${\mathrm{SpO}}_{2}$. From the absorption spectra of melanin, the 690 nm has higher absorption than 830 nm. This wavelength-dependent behavior of melanin absorption was found to be critical in mitigating its effects on optical measurements.^59^ We observed a significant negative correlation between the melanin index and SNR at 690 nm, as opposed to 830 nm, highlighting the critical role of wavelength selection. We extended this analysis to the investigation of ${\mathrm{SpO}}_{2}$, and Fig. S9 in the Supplementary Material shows that the ratio R increases significantly with the melanin index, which mathematically lowers the ${\mathrm{SpO}}_{2}$ values. Our findings are consistent with the existing literature that suggested melanin absorption can have wavelength-dependent behavior, which is an important factor to consider in mitigating these effects.^59^

In contrast, our study found that the FDMD method demonstrates limited change in ${\mathrm{StO}}_{2}$, $\mu_{a}$, and $\mu_{s}^{\prime }$ for participants with melanin variations. This robustness is attributed to how FDMD calculates the [HbO] and [Hb] by analyzing the slope values of AC or DC intensities and phase against the source–detector distance, which maintain consistency across different melanin indices.^60^ Thus, the impact of melanin on signal quality is uniformly distributed across all source–detector distances for a given participant. This observation aligns with the findings of Franceschini et al.,^61^ who explored the influence of superficial layers on two-layered turbid media and concluded that the efficacy of the FDMD method for thin first layers stays effective regardless of superficial layers.

Our findings highlight the importance of carefully selecting the measurement wavelengths to ensure accurate and reliable results, particularly when measuring individuals with darker skin tones. Further research work is required to understand how the wavelength selection can elaborate our understanding of the NIRS bias characteristic. However, ${\mathrm{StO}}_{2}$, $\mu_{a}$, and $\mu_{s}^{\prime }$ measurements are reliable despite melanin variation in different clinical settings.

### 4.3 Research Question 3

The fundamental principle of the FD-NIRS system helps interpret the effect of melanin on the NIRS signal. Melanin in the skin absorbs more 690 nm photons than 830 nm DC intensities, which results in increased photons from 830 nm ($r_{s}=0.506$, p=0.004) and decreased 690 nm DC intensity ($r_{s}=-0.491$, p=0.006) as the melanin index increases. In the Oxiplex system, we do not control the input intensity adjustment. The device adapts the detector gain based on the total photon, Fig. 5. As an example, the total photon count for participants with a melanin index of 32 has a distribution where 60% of the photons are from 830 nm, and 40% are from 690 nm. For the participants with a melanin index of 100, 90% of the total photon count comes from 830 nm and 10% from 690 nm. FD-NIRS systems use frequency-modulated intensities and do not rely on calibration methods to quantify ${\mathrm{SpO}}_{2}$, yet the differences in light scattering lead to inaccurate ${\mathrm{SpO}}_{2}$ values. Because of this operating principle, it would not be accurate to calculate ${\mathrm{SpO}}_{2}$ from mBLL, and we found it important to determine ${\mathrm{SpO}}_{2}$ based on R. Using similar methods to pulse oximetry, we found that the ratio R [Eq. (S2) in the Supplementary Material] increases significantly with the melanin index. Our findings suggest that future research can implement input intensity adaptation based on the participant’s melanin index.

### 4.4 Limitations

The absence of arterial blood gas measurements precludes a direct correlation with the melanin index, leaving a gap in the physiological validation of our findings. Furthermore, our study did not encompass participants with lower-than-normal ${\mathrm{SpO}}_{2}$ ranges, which could have provided insights into the effects of occult hypoxemia on the metrics observed. The investigation was also limited to a resting state, omitting the potential variability introduced by functional tasks, which could affect hemodynamic data and possibly reveal different bias characteristics. Our reliance on an FD-NIRS system, which uses a laser-based photon source system, may only partially represent the outcomes that could be observed with LED-based sources as recent literature suggested potential differences in measurement outcomes.^62^ In addition, the use of a device with only two wavelengths limits our ability to assess whether additional wavelengths could mitigate the impact of melanin on NIRS measurements. These constraints highlight the necessity for broader research incorporating varied physiological states, measurement technologies, and participant demographics to understand melanin’s implications on optical measurements comprehensively.

## 5 Conclusion

In this study, we presented a robust, quantifiable approach for characterizing the effect of melanin and the causation of bias on NIRS signals in a diverse, healthy participant population. Our findings clearly demonstrate a statistically significant negative correlation between melanin index and SNR, particularly at the wavelength of 690 nm. This suggests that participants with higher melanin content experience lower SNR values, primarily due to the increased noise associated with higher melanin absorption. Furthermore, the study reveals a significant negative correlation between ${\mathrm{SpO}}_{2}$ levels and the melanin index. This correlation is driven by the wavelength-dependent absorption properties of melanin, which disproportionately affect the distribution of photon counts between the wavelengths of 830 and 690 mm. This imbalance, in turn, impacts the calculation of ${\mathrm{SpO}}_{2}$, highlighting the importance of considering melanin’s optical properties in the design and interpretation of NIRS measurements. Conversely, ${\mathrm{StO}}_{2}$ measurements, as well as absorption and reduced scattering coefficients, show no significant correlation with the melanin index. This suggests that certain NIRS metrics may be less susceptible to the effects of melanin potentially due to the methodologies employed for their calculation, such as the multi-distance approach, which appears to mitigate the influence of melanin on signal quality. The research further underscores the critical role of the operating principles of FD-NIRS devices in addressing discrepancies related to skin pigmentation. The study emphasizes the importance of considering the optical properties of melanin in NIRS technology development and application, advocating for adjustments in device operation, such as the Oxiplex system’s detector gain adjustment, to ensure accurate, inclusive outcomes for all skin types.

## Supplementary Material



## Data Availability

The data that support the findings of this article are not publicly available due to privacy concerns. They can be requested from Sossena Wood at scwood@andrew.cmu.edu.
