# Supplementary material for: Exploring the impact and influence of melanin on frequency-domain near-infrared spectroscopy measurements
Source: J Biomed Opt. 2024 Sep 25;29(Suppl 3):S33310. doi: 10.1117/1.JBO.29.S3.S33310 (PMC11423252; doi:10.1117/1.JBO.29.S3.S33310)
Supplement: Supplementary file 1 [file JBO_029_S33310_SD001.pdf]

Supplementary Material

The data in this supplemental material are to support our study's three research questions and findings. It will also present supporting material for the Differential Pathlength Factor (DPF) calculation and alternate representation of SpO_2.

Method

Matthias et al. provide the foundation for our study of DPF determination, which investigates the dependence of the normalized DPF on wavelength [48]. To calculate the DPF ratio used in our study, we specifically extracted normalized DPF values at the used wavelengths: 690 nm and 830 nm. The normalized DPF at 690 nm was determined using a cubic extrapolation method. This technique was chosen because it can capture the underlying structure of the DPF values at lower wavelengths. Meanwhile, for the normalized DPF at 830 nm, we employed interpolation.

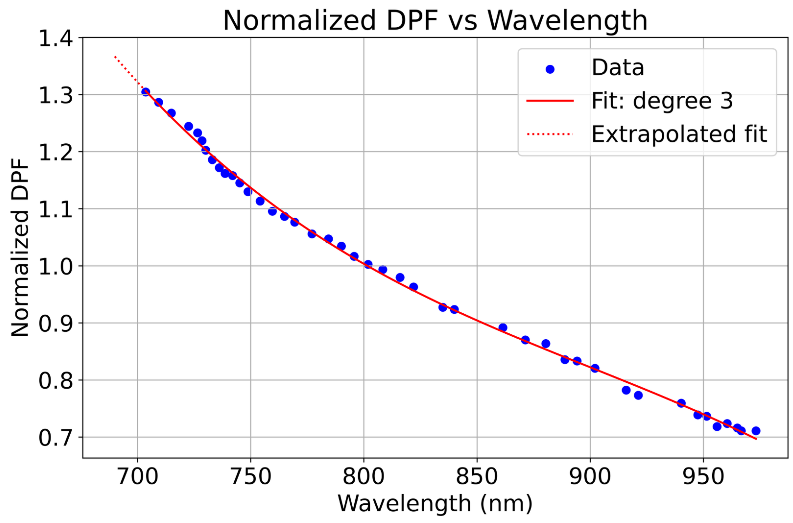

Fig. S1. Normalized DPF variation as a function of wavelength. The blue dots represent experimental data extracted from the literature [48]. The red line is a cubic fit to the data, exhibiting a high degree of fit with an R^2 value of 0.9981.

Figure S1 visualizes this variation of the normalized DPF with wavelength. The experimental data points, depicted as blue dots, have been derived from the literature. A cubic fit (shown as the red line) was performed on this data, yielding an extremely high R^2 value of 0.9981, indicating that the cubic model provides a remarkably accurate fit to the observed data.

Figure S3 show the spectrograms for Δ[HbO] and for Δ[Hb] (derived from mBLL using the calculated DPF ratio). The spectrogram for a 60-second data with a window span of 20 seconds, with a 95% overlap. The maximum-energy time-frequency ridge power associated with the heart rate was extracted from the Δ[HbO] signal using the "tfridge" function of Matlab with a penalty of 0.005. These extracted power values have been used as $\Delta[\text{HbO}]_{HR}$ and for $\Delta[\text{Hb}]_{HR}$ in equation 4.

Melanin index measurement

Melanin index calculation is based on the diffuse reflectance in the red spectrum centered at 680 nm, where melanin is the predominant absorbing chromophore, and the interference from hemoglobin is minimal. The melanin index (M) is calculated as follows:

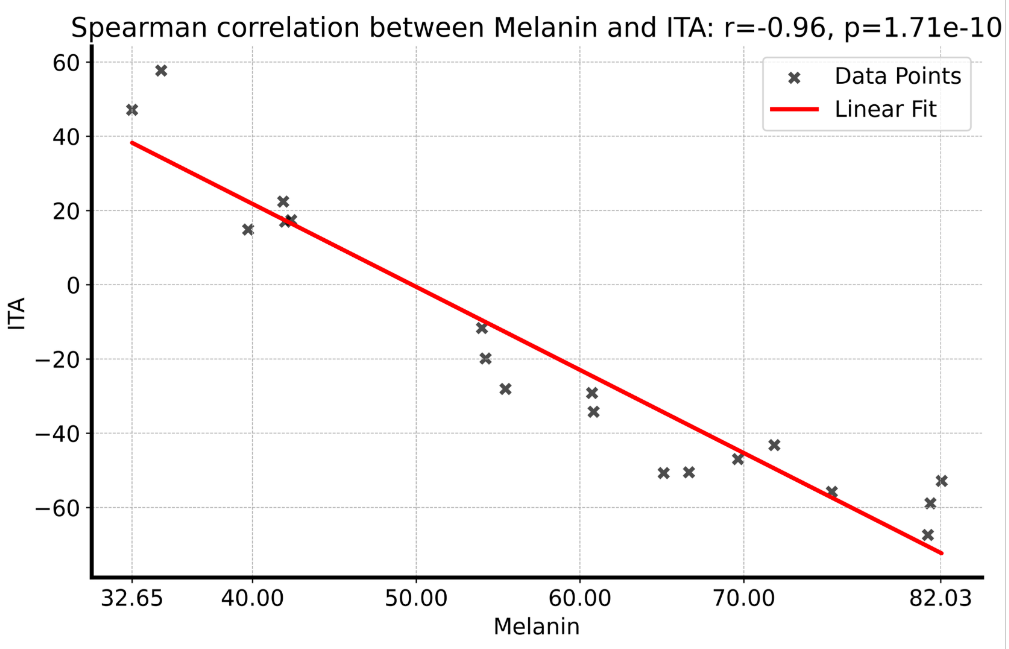

Fig. S2. For a sub-cohort of our participants (n=19), we found that both ITA and melanin index measurements are highly correlated to one another (r=-0.96, p=1.71e-10). This helps us to make reasonable assumptions that ITA and melanin can be used interchangeably.

$$M = 100 \times \log_{10}\left(\frac{1}{R_r}\right)$$

where M is the melanin index, and R_r the reflectance is at 680 nm.

Alternate representation of SpO_2

To isolate the impact from different wavelength photons for the SpO_2 analysis, we can use this expression to calculate SpO_2 values [63]-

$$SpO_2 = \frac{\epsilon_{Hb}^{\lambda_2} R(B^{\lambda_2}/B^{\lambda_1}) - \epsilon_{Hb}^{\lambda_1}}{\left(\epsilon_{HbO}^{\lambda_1} - \epsilon_{Hb}^{\lambda_1}\right) - R(B^{\lambda_2}/B^{\lambda_1})\left(\epsilon_{HbO}^{\lambda_2} - \epsilon_{Hb}^{\lambda_2}\right)} \tag{S1}$$

The extinction coefficients are fixed value constants independent of melanin [49]. B is a wavelength-dependent factor that can be determined from the figure S1. Further, the ratio R is calculated with this formula -

$$R = \frac{\frac{AC690}{DC690}}{\frac{AC830}{DC830}} = \frac{AC690}{AC830} \times \frac{DC830}{DC690}, \tag{S2}$$

where AC intensity refers to the pulsatile component of the light absorbed, which is associated with the arterial blood pulse. DC refers to the non-pulsatile component of the light absorbed. From this expression, the two variables that can impact SpO_2 are B and R.

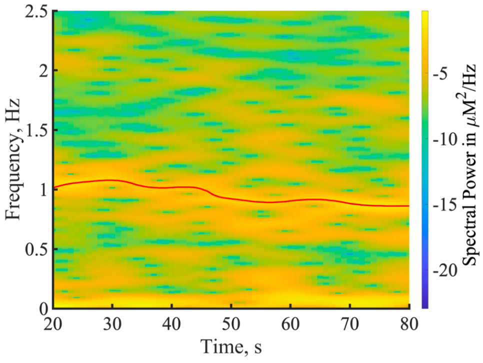

(a) Δ[HbO] signal

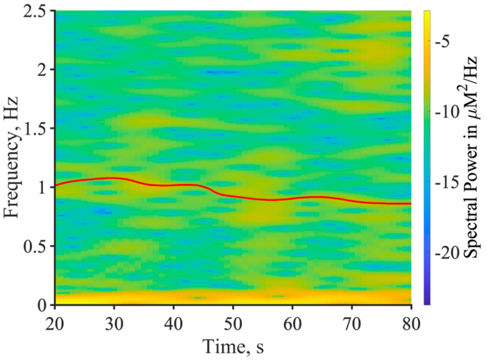

(b) Δ[Hb] signal

Fig. S3. In the spectrograms, the red line marks the maximum-energy time-frequency ridge power associated with the heart rate extracted from the Δ[HbO] signal.

Result and Discussion

Research Question 1

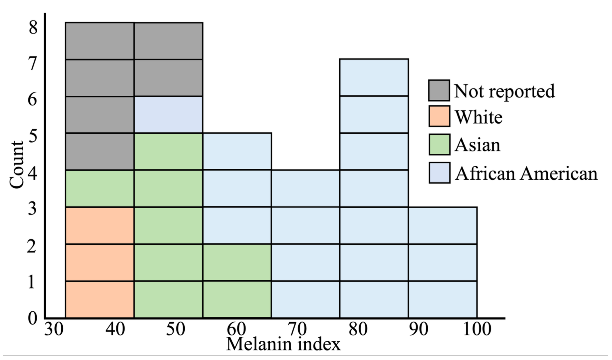

Fig. S4. The self-reported race shows multiple melanin indexes within a given race. There are six participants who did not report race.

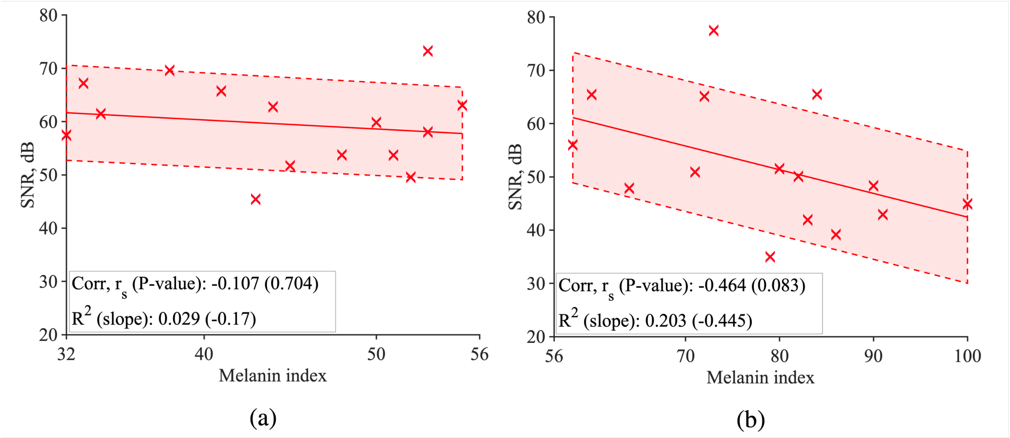

Fig. S5. SNR estimation for the participants with melanin indices (a) for the first 15 participants (melanin indices 32 to 55) (b) and the last 15 participants (melanin indices 58 to 100) shows the SNR estimation decrease is more pronounced for the last 15 participants.

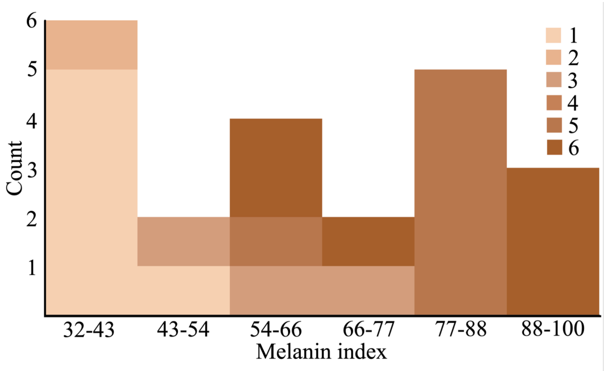

Fig. S6. The Fitzpatrick scale can be misleading to quantify the melanin index of a participant. The self-reported Fitzpatrick scale shows participants tend to rate themselves as darker than their respective melanin index.

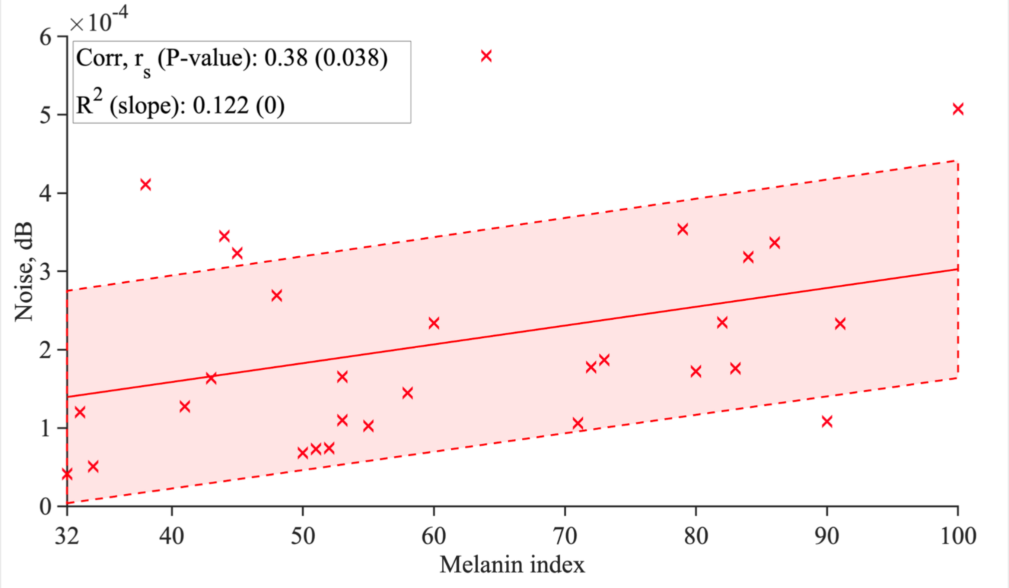

Fig. S7. Noise estimation shows a significant positive correlation with the melanin index (r_s = 0.38, p = 0.038).

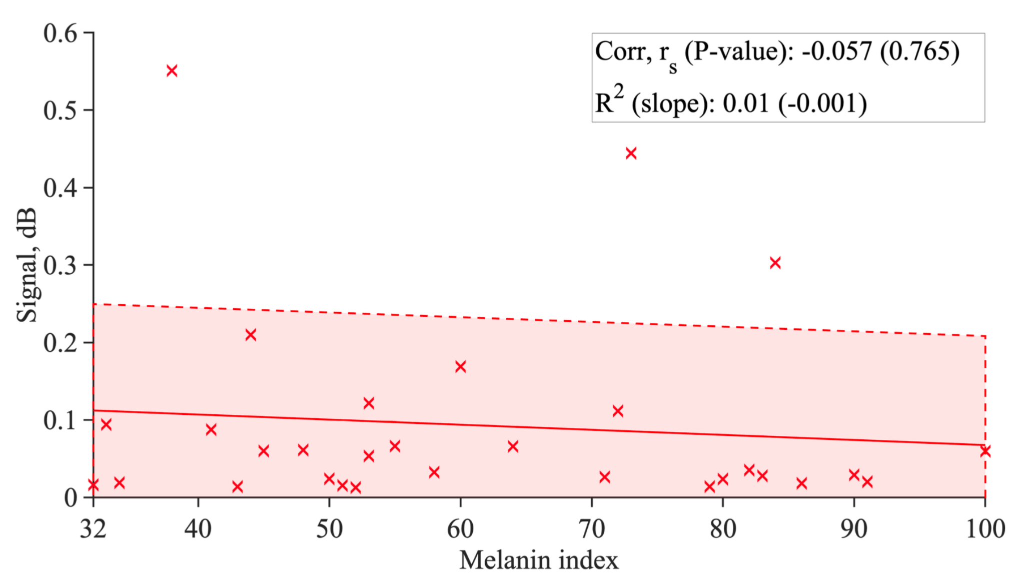

Fig. S8. Signal estimation shows no significant correlation with the melanin index (r_s = 0.057, p = 0.765).

Research Question 3

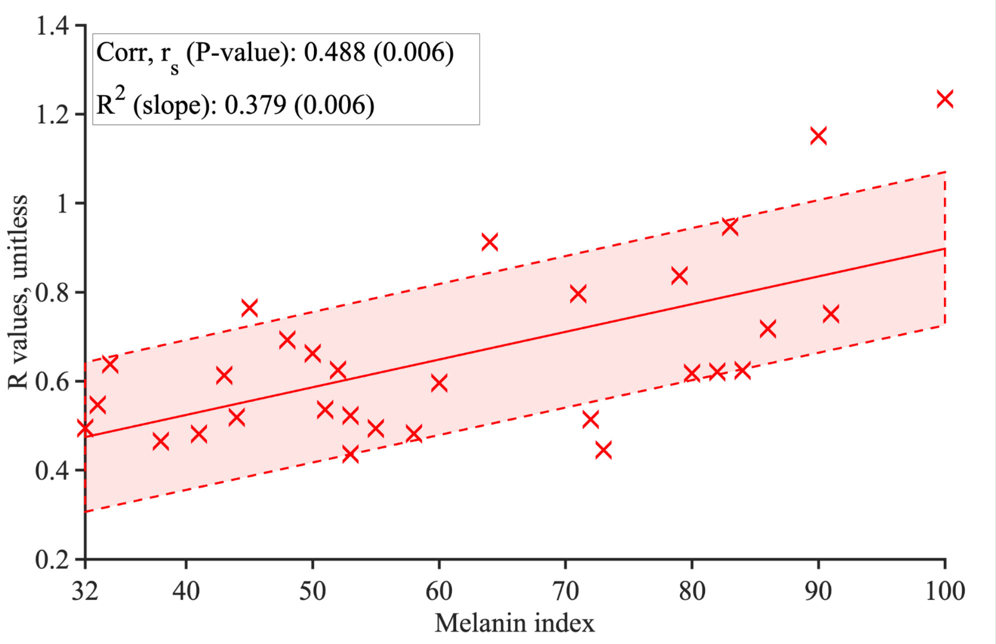

Fig. S9. The ratio, R, has a significant positive correlation with melanin index ($r_s = 0.488$, $p = 0.006$).

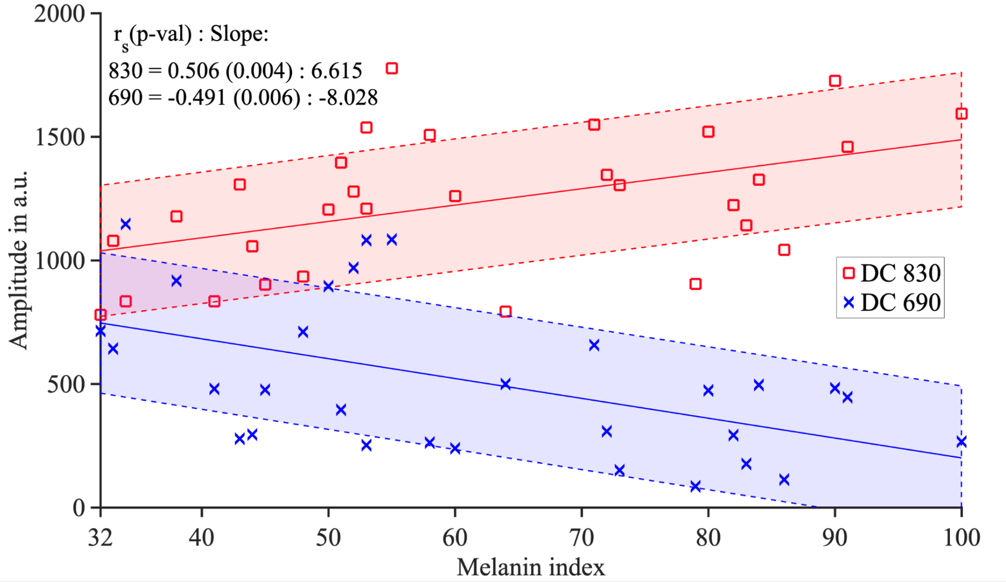

Fig. S10. DC comparison from both wavelengths show the result of the detector gain from figure 5. Because of the detector gain, the 830 nm intensity increases ($r_s = 0.506$, $p = 0.004$), and the 690 nm intensity decreases significantly ($r_s = -0.491$, $p = 0.006$).

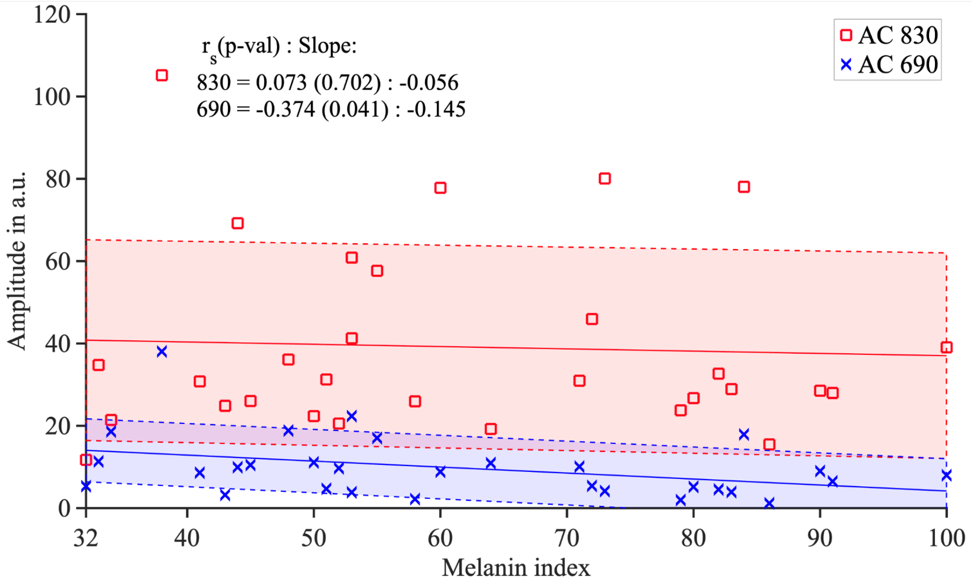

Fig. S11. The excess 830 nm photon count does not result in more cardiac pulse detection capability ($r_s = 0.073$, $p = 0.702$). At 690 nm, on the other hand, the decrease of photon count decreases in the cardiac detection capability ($r_s = -0.374$, $p = 0.041$).
